# Care Outcomes for Chiropractic Outpatient Veterans (COCOV): a single-arm, pragmatic, pilot trial of multimodal chiropractic care for U.S. veterans with chronic low back pain

**DOI:** 10.1186/s40814-022-01008-0

**Published:** 2022-03-07

**Authors:** Cynthia R. Long, Stacie A. Salsbury, Robert D. Vining, Anthony J. Lisi, Lance Corber, Elissa Twist, Thad Abrams, Robert B. Wallace, Christine M. Goertz

**Affiliations:** 1 Palmer Center for Chiropractic Research, Davenport, Palmer College of Chiropractic, 741 Brady St, Davenport, IA 52803 USA; 2 Yale Center for Medical Informatics, Yale University, New Haven, CT USA; 3 Department of Internal Medicine, University of Iowa, Iowa City VA Health Care System, Center for Access & Delivery Research and Evaluation, Iowa City, IA USA; 4 Department of Epidemiology, College of Public Health, University of Iowa, Iowa City, IA USA; 5 Department of Orthopaedic Surgery, Duke University School of Medicine, Durham, NC USA

**Keywords:** Low back pain, Veterans, Pain management, Chiropractic, Patient-reported outcome measures, Pilot projects, Chronic pain, Nonpharmacologic

## Abstract

**Background:**

Over 25% of veterans seeking care at U.S. Veterans Health Administration facilities have chronic low back pain (LBP), with high rates of mental health comorbidities. The primary objective of this study was to assess the feasibility of participant recruitment, retention, and electronic data collection to prepare for the subsequent randomized trial of multimodal chiropractic care for pain management of veterans with chronic low back pain. The secondary objectives were to estimate effect sizes and variability of the primary outcome and choose secondary outcomes for the full-scale trial.

**Methods:**

This single-arm pilot trial enrolled 40 veterans with chronic LBP at one Veterans Health Administration facility for a 10-week course of pragmatic multimodal chiropractic care. Recruitment was by (1) provider referral, (2) invitational letter from the electronic health record pre-screening, and (3) standard direct recruitment. We administered patient-reported outcome assessments through an email link to REDCap, an electronic data capture platform, at baseline and 5 additional timepoints. Retention was tracked through adherence to the treatment plan and completion rates of outcome assessments. Descriptive statistics were calculated for baseline characteristics and outcome variables.

**Results:**

We screened 91 veterans over 6 months to enroll our goal of 40 participants. Seventy percent were recruited through provider referrals. Mean age (range) was 53 (22–79) years and 23% were female; 95% had mental health comorbidities. The mean number of chiropractic visits was 4.5 (1–7). Participants adhered to their treatment plan, with exception of 3 who attended only their first visit. All participants completed assessments at the in-person baseline visit and 80% at the week 10 final endpoint. We had no issues administering assessments via REDCap. We observed clinically important improvements on the Roland-Morris Disability Questionnaire [mean change (SD): 3.6 (6.1)] and on PROMIS® pain interference [mean change (SD): 3.6 (5.6)], which will be our primary and key secondary outcome, respectively, for the full-scale trial.

**Conclusions:**

We demonstrated the feasibility of participant recruitment, retention, and electronic data collection for conducting a pragmatic clinical trial of chiropractic care in a Veterans Health Administration facility. Using the pilot data and lessons learned, we modified and refined a protocol for a full-scale, multisite, pragmatic, National Institutes of Health-funded randomized trial of multimodal chiropractic care for veterans with chronic LBP that began recruitment in February 2021.

**Trial registration:**

ClinicalTrials.gov NCT03254719

**Supplementary Information:**

The online version contains supplementary material available at 10.1186/s40814-022-01008-0.

## Key messages regarding feasibility

- Chiropractic services are provided at U.S. Veterans Affairs healthcare facilities, but the feasibility of conducting pragmatic randomized trials of multimodal chiropractic care for veterans with chronic low back pain in this setting was unknown.
- We demonstrated the feasibility of participant recruitment, retention, and electronic data collection in one Veterans Affairs healthcare facility. We also estimated effect sizes for the primary outcomes and selected secondary outcomes for use in the full-scale trial.
- Using the pilot data and lessons learned, we modified and refined a protocol for a full-scale, multisite, pragmatic, National Institutes of Health-funded randomized trial of multimodal chiropractic care for veterans with chronic low back pain that began recruitment early in 2021.

## Background

Over 50% of veterans receiving care in U.S. Department of Veterans Affairs, Veterans Health Administration (VA) facilities are diagnosed with a musculoskeletal condition, 25% of whom have chronic back pain [1, 2]. In older veterans, factors associated with high pain-related disability are low back pain (LBP), high pain intensity, and depressive symptoms [3]. Veterans with musculoskeletal pain report high rates of depression, anxiety, post-traumatic stress disorder (PTSD), and alcohol use, which are increasingly being diagnosed in younger veterans [1]. Female veterans with pain report higher pain intensity and are more likely to report PTSD, depression, and anxiety than male veterans [4]. Veterans in pain are often managed using overlapping pain and mental illness prescription drugs, including opioids, psychotropic medications, and sleep agents [2].

The VA has implemented policies to improve clinical pain management, including the recommendation of nonpharmacological approaches where sufficient evidence of safety and effectiveness is found [5–7], such as spinal manipulation and multimodal chiropractic care for patients with LBP [8, 9]. In 2004, VA expanded its delivery of nonpharmacological treatment options for veterans with musculoskeletal conditions by providing chiropractic services, which are part of the standard medical benefits package available to veterans [10]. Chiropractic care is currently integrated with primary care, rehabilitation, pain management, and other specialty teams in 151 VA healthcare facilities [11]. In VA Fiscal Year 2019, over 66,000 veterans received chiropractic care [12].

Veterans access VA chiropractic services through referral from a VA primary care or specialty provider. Doctors of chiropractic (DCs) in VA provide diagnosis and management of non-surgical musculoskeletal and neuromuscular conditions, most often related to the low back and neck. Treatment options of multimodal chiropractic care include patient education, active rehabilitation, spinal manipulation and other manual therapies, and self-management advice [13, 14].

Mechanisms of multimodal chiropractic care are not fully understood. However, current evidence suggests spinal manipulation can initiate neurophysiological changes resulting in reduced pain [15, 16], disrupt intra- and peri-articular joint adhesions, and improve joint or regional mobility in the spine [17–19]. Specific exercises are used to reduce abnormal mechanical loading of spinal structures from weak or poorly coordinated muscle groups [20]. Other active treatments are used to improve mobility, reduce fear of movement, and provide scaffolded activities supporting self-efficacy and improved function [21, 22]. Education about a condition, especially chronic pain, is designed to help patients understand and better interpret symptoms, leading to greater self-monitoring and self-management capacity [23–25].

Although VA chiropractic services have grown considerably over the past 15 years, few studies have prospectively evaluated chiropractic care for pain and disability in veterans [26, 27]. To prepare for a future multisite, pragmatic randomized trial in VA healthcare environments, our team conducted a mixed method, single-arm, pragmatic, pilot clinical trial of multimodal chiropractic care for veterans with chronic LBP. The primary objectives of this pilot study were to assess the feasibility of participant recruitment, retention, and electronic data collection methods. Secondary objectives were to estimate effect sizes and variability of the outcome measures and select secondary outcomes for use in the full-scale trial.

## Methods

### Study design and setting

This single-arm, pragmatic, pilot clinical trial was conducted at the Iowa City Veterans Affairs Health Care System in the Iowa City, IA VA hospital and Coralville, IA Community-Based Outpatient Clinic. Chiropractic care was administered by licensed, credentialed, and privileged DCs employed by the Iowa City VA. The study is reported according to the CONSORT extension to pilot and feasibility trials guidelines (Additional file 1) [28].

### Ethical considerations

The trial was approved by The University of Iowa IRB-03 VA Only, the VA Connecticut Health System IRB, and the Palmer College of Chiropractic IRB. The trial was overseen by an independent data and safety monitoring committee. All participants provided written informed consent. Participants were compensated with a $25 gift card after completing outcome assessments at the baseline visit, week 5, and week 10 with an additional $25 gift card for completing all 3 assessments. They also received trial-supported travel reimbursements for driving distances beyond 20 miles.

### Participants

U.S. veterans aged 18 years and older reporting chronic LBP, defined as LBP persisting ≥ 3 months with pain on at least half the days in the past 6 months [29], with or without mental health comorbidities were eligible. Receiving any chiropractic care within the past 90 days, impaired cognitive ability, contraindications to chiropractic care, inability to attend chiropractic appointments per recommended treatment plan, an active suicide monitoring flag in the electronic health record (EHR), and need for a proxy to complete questionnaires were exclusionary.

We targeted a sample size of 40 participants with a goal of enrolling 6–8 participants per month over a 6-month period. Research has historically only included women in proportion to their VA representation, which is currently 10% [30]. However, we aimed to oversample women, who currently comprise 15.8% of patients seen in VA chiropractic clinics annually [31], with a goal of enrolling 20% or more female veterans.

### Participant recruitment

Three different recruitment strategies were tested. For the first strategy, team members shared study information with VA primary care physicians and nurse case managers to promote referrals, including regular contact with the Iowa City VA Women’s Health Clinic. The DC at the Iowa City VA hospital also screened the list of new patients at least weekly and shared the names of those possibly eligible with the study coordinator. The second strategy used a list created from the VA EHR of patients who had a visit to a primary care provider at the Iowa City VA with an International Classification of Diseases, edition 10 (ICD-10) code for LBP (see Additional file 2) within the past year who did not see a DC; name, address, sex, race, ethnicity, and age were included on the list. To encourage women and minority veterans to consider participating in the research study, we sent letters to all females (*n*=69) and non-white and Hispanic males (*n*=32) living within 50 miles of the Iowa City VA. We also sent letters to a subset of white males within 25 miles of the Iowa City VA (*n*=101). The materials created for the standard direct recruitment strategy were placed in the Iowa City VA and area veteran organizations in coordination with the Iowa City VA public affairs office. The study coordinator contacted the patients identified through all 3 strategies by phone to ascertain their interest in the study.

### Participant screening

The study coordinator conducted a phone screen for veterans who were interested in the trial. After verbal consent, potential participants were asked 7 screening questions including confirmation they were a veteran who received care at the Iowa City VA and had chronic LBP. Those remaining eligible and interested were scheduled for a baseline visit before their first visit to the chiropractic clinic. During the baseline visit, veterans were guided through the informed consent process followed by a brief screening interview. Those who remained eligible then completed the baseline questionnaires. The DC completed the final screen after the first visit with the veteran to determine if the patient had a diagnosis of LBP appropriate for a course of chiropractic care.

### Study interventions

Chiropractic care decisions were based on routine work-up, including in-office clinical evaluation, health history and record review, and other diagnostic tests when indicated. Clinical evaluation screened for pathology requiring additional testing or referral and informed working diagnoses. Care plans including visit frequency and duration were determined individually based on factors such as working diagnoses, participant preferences, symptom duration and severity, response to prior care, and the presence of comorbidities.

DCs monitored participant health status throughout the trial with the capacity to initiate referrals when clinically indicated. Multimodal chiropractic care typically included some form of thrust or non-thrust spinal manipulation and other interventions such as education, rehabilitative exercise, stretching, and self-management advice based on factors unique to each case, including participant goals and preferences. The integrated care pathway developed prior to this study was made available to the DCs [13]. Care was provided during office visits for a duration of up to 10 weeks, with a minimum treatment frequency of 1 visit and a maximum of 12 visits. Outside of chiropractic care, participants could access the same medical and mental health care available to all veterans during the study timeframe. Participants were not asked to limit care with 2 exceptions: avoid chiropractic care outside the VA and avoid acupuncture from VA chiropractors.

### Blinding

DCs providing clinical care, study coordinators, and all investigators were blinded to study outcome measures.

### Data collection

The data collection schedule is shown in Table 1. The Yale Center for Medical Informatics developed, programmed, and implemented the EHR data extraction queries and methods for obtaining participant data for recruitment efforts, as well as demographic variables. The Palmer Center for Chiropractic Research developed, programmed, and implemented the patient-reported outcome assessments that were administered at baseline, after baseline before the first DC visit, after the first DC visit and at weeks 3, 5, 7, and 10 via electronic data capture by REDCap (Research Electronic Data Capture, Vanderbilt University, Nashville, TN). We split the baseline measures into 2 components, one to be completed during the in-person baseline visit and the other after the baseline visit before the first DC visit, to lessen participant burden. The results of the qualitative interview completed as part of the data collection process are reported in a companion article.

**Table 1:** Outcome assessment schedule

Questionnaire	EHR	Baseline visit		Week 3	Week 5	Week 7	Week 10	Use in a full-scale trial
		In-person	After					
Demographics	X	X						X
RMDQ		X			X		X	X
Brief Pain Inventory		X			X		X	
PHQ-9		X					X	X
GAD-7		X					X	X
PLC-C		X					X	X
AUDIT		X					X	X
PASTOR items								
DVPRS			X		X		X	
PROMIS®								
Global health			X		X		X	X
Neuropathic pain			X					X
Pain interference			X		X		X	X
Physical function			X		X		X	X
Fatigue			X		X		X	X
Sleep			X		X		X	X
Depression			X		X		X	
Anxiety			X		X		X	
Anger			X		X		X	X
Alcohol use			X		X		X	
Satisfaction with social role			X		X		X	X
Self-efficacy for managing symptoms			X				X	X
Other PASTOR questions			X		X		X	
EXPECT			X					X
LBP self-care			X				X	X
PEG				X		X		X
HEAL			X^a^				X^b^	X
Adverse events					X		X	X
Qualitative interview							X^c^	X

*Abbreviations*: *RMDQ* Roland-Morris Disability Questionnaire, *PHQ-8* Patient Health Questionnaire, *GAD-7* Generalized Anxiety Disorder, *PCL-C* PTSD Checklist-Civilian, *AUDIT-10* Alcohol Use Disorders Identification Test, *PASTOR* Pain Assessment Screening Tool and Outcomes Registry, *DVPRS* Defense & Veterans Pain Rating Scale, *EXPECT* Expectations for Complementary and Alternative Medicine Treatments, *LBP* low back pain, *PEG* Pain, Enjoyment of life, and General activity, *HEAL* Healing Encounters and Attitudes List

^a^Administered following initial chiropractic visit

^b^Administered prior to the last scheduled visit

^c^Administered from 8 to 38 weeks

Participants used an on-site study-provided laptop to link to and complete the REDCap baseline assessment. An automated email reminder to complete each assessment after the baseline visit was sent to participants via REDCap the first and last days of the 7 days the assessment window was open to access their REDCap form on their home or public access devices. For weeks 5 and 10, the study coordinator contacted the participant via voice or text message at least once during this window to remind the participant to complete the assessment. Once the active window closed for the week 5 and 10 assessments, the study coordinator attempted to contact the participant by phone up to 3 times within 7 days to complete a computer-assisted telephone interview (CATI). Our feasibility goal for completed outcome assessments at the week 10 primary endpoint was at least 80%.

### Outcomes

The primary outcome measure, the modified 24-item version of the Roland-Morris Disability Questionnaire (RMDQ), was chosen for the full-scale trial. RMDQ is a one-page questionnaire to measure LBP-related disability with documented reliability, validity, and sensitivity to clinical change [8, 32]. Other recent clinical trials of nonpharmacologic therapies for veterans with chronic LBP have used the RMDQ as the primary outcome [33].

We administered a battery of questionnaires, described below, to aid in determining the secondary outcomes to collect during the full-scale trial. We also tracked the time used to complete each assessment.

The Brief Pain Inventory (BPI) assessed pain intensity and pain interference over the past 24 h. The BPI was developed to assess pain associated with cancer, but is now also used for noncancer pain [34, 35]. The scored items of the survey include 4 questions to rate pain intensity using a 0–10 numeric rating scale (NRS). The final 7 questions use a 0–10 NRS to rate how pain interferes with activities of daily life, mood, walking ability, normal work, relationships, sleep, and enjoyment of life. An overall pain interference measure is calculated as the mean over the 7 items.

The Pain, Enjoyment, and General Activity (PEG) is a 3-item tool for chronic pain assessing average pain intensity, interference with enjoyment of life, and interference with general activity over the past week [36]. We revised PEG to ask about back pain rather than chronic pain. We collected PEG at weeks 3 and 7 to pilot the tool via email for use via weekly text messaging in the full-scale trial.

Evidence-based legacy instruments used in VA were administered to measure depression, anxiety, PTSD, and alcohol use. The Patient Health Questionnaire (PHQ-9) has 9 items assessing depressive disorder [37]. Total scores range from 0 to 27 with a score of 10–14 considered to be in the moderate range. PHQ-9 has a test-retest reliability of 0.81 to 0.96 in primary care settings. Generalized anxiety disorder (GAD) was assessed by the 7-item GAD [38]. At a cutoff score of 10, GAD-7 has a sensitivity of 0.89 and specificity of 0.82 for identifying patients with GAD in primary care settings. The PTSD Checklist-Civilian (PCL-C) has 17 items [39–41]. A total severity score is determined by summing over the items with a change of 5–10 points representing the minimum threshold for determining treatment response; a 10–20 point change represents a clinically important improvement in PTSD symptom severity. The Alcohol Use Disorders Identification Test (AUDIT) has 10 items screening for harmful or hazardous alcohol consumption [42].

We also administered the Pain Assessment Screening Tool and Outcomes Registry (PASTOR), a data collection tool for chronic pain adopted by the Department of Defense/VA Pain Management Task Force [43]. Specific measures included the Defense & Veterans Pain Rating Scale (DVPRS), an 11-item (0–10) numeric rating scale (NRS) that includes a Faces Rating Scale component and 4, 0–10 NRS items to quantify the impact of pain on general activity, sleep, mood, and stress [44]. An overall pain interference measure was calculated as the mean over the 4 items. PASTOR also included 2 additional NRS measures for pain on a 1–10 scale, with words describing each level.

PASTOR incorporated Patient-Reported Outcome Measurement Information System (PROMIS®) measures for mental health conditions (depression, anxiety, sleep disturbance, anger, and alcohol use), physical and social function, fatigue, and pain interference. We used the Assessment Center^SM^ Application Programming Interface to administer computer adaptive testing for these PROMIS® domains [45]. PASTOR also included the PROMIS® domains for global pain, neuropathic pain, and efficacy for managing symptoms [46]. The Assessment Center calculated all domain-specific *T*-scores, which were normed to a mean of 50 and SD of 10 based on the 2000 U.S. general census [45]. PASTOR had additional questions on pain treatment history, headaches, PTSD, and current medication and opioid use. PASTOR provides a 3-page Clinician Report on these measures that was not used in this pilot trial.

The Expectations for Complementary and Alternative Medicine Treatments (EXPECT) questionnaire was developed to assess individuals’ expectations of back pain treatments [47]. We asked participants about their expectations of the effect that chiropractic care would have on their LBP. The Healing Encounters and Attitudes Lists (HEAL) is a validated item-bank comprised of 6 domains developed through PROMIS® methodology [48]. We used HEAL to assess nonspecific factors known to influence patient outcomes, including perceptions of the doctor-patient relationship [49–55], treatment expectancy [56, 57], and positive or negative outlook [58]. We asked participants to respond to these questions about their current VA chiropractor. A back pain self-care questionnaire that assessed 18 self-care strategies was developed from previous studies that collected patient-reported self-care activities [59, 60].

### Adverse events

Participants were instructed to contact the study coordinator if they experienced a change in health status or significant pain, discomfort, or distress during the study duration. DCs were asked to contact the study coordinator if a patient reported any of these issues. Participants were also asked to inform the study coordinator of any unplanned emergency room visits or hospitalizations. We inquired about adverse events at weeks 5 and 10 using a questionnaire asking “Did you experience any discomfort or unpleasant reaction after any of your chiropractic treatments?” “Yes” responses were followed by indicating a category (e.g., increased pain, stiffness, muscle weakness, headache). The severity of symptoms was rated by indicating how much a symptom required changing or modifying regular activities, including life-threatening or required hospitalization. Study coordinators were alerted to follow-up on the latter 2 and work with a central trial DC to ascertain if it was a serious adverse event requiring reporting to principal investigators, our funding organization, and IRBs.

### Sample size

We sought to enroll 40 participants over a 6-month duration. In order to adequately assess the feasibility of our recruitment methods (including the ability to enroll at least 20% females and recruit across the lifespan) and retention methods, test the use of electronic data capture for patient-reported outcomes, and choose secondary outcome measures for the full-scale trial, we chose a higher than typical sample size.

### Data analysis

Descriptive statistics were calculated for all baseline characteristics and outcome variables using SAS 9.4 (SAS Institute Inc., Cary, North Carolina). Consistent with recommendations for pilot studies [28, 61], the study was not powered to detect clinical effectiveness and no statistical testing was conducted.

## Results

**Fig. 1:**
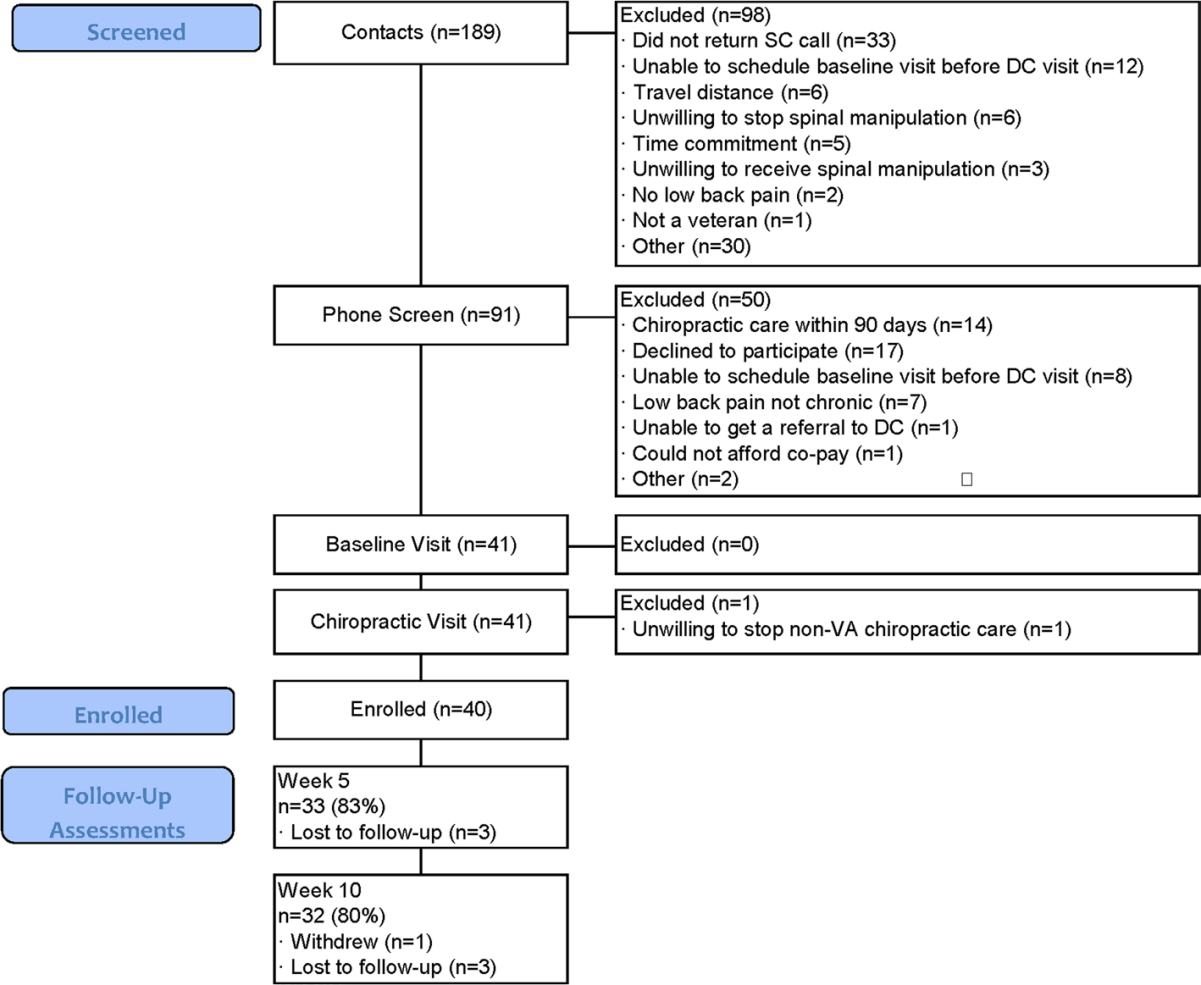
Trial flow

A total of 91 veterans were screened between February 19 and August 27, 2018, with 40 participants enrolled in the trial (Fig. 1). Data collection was completed on November 5, 2018. The mean (range) participant age was 53 years (22–79), 9 participants (23%) were female, and 3 participants (7.5%) were non-white or Hispanic (Table 2). All participants had chronic LBP, with 33 (83%) experiencing LBP every day in the past 6 months. Thirty-eight (95%) participants had mental health comorbidities, but few had alcohol use disorder, with 73% reporting monthly or less alcohol use. Nine (23%) participants reported the use of nonsteroidal anti-inflammatory drugs, 7 (18%) neuropathic pain medications such as gabapentin, and 4 (10%) opioids for their LBP. Participants reported high hopes for change in back pain and the impact of back pain on life, but far lower realistically expected levels of change (Table 2).

**Table 2:** Baseline characteristics (*n* = 40)

Age, mean (range), y	53.3 (22–79)
Age group, *n* (%)	
18–35 years	6 (15)
36–54 years	16 (40)
55+ years	18 (45)
Sex, *n* (%)	
Male	31 (78)
Female	9 (23)
Hispanic or Latino, *n* (%)	2 (5)
White race, *n* (%)	36 (90)
History of mental health diagnosis identified in EHR, *n* (%)	29 (73)
Patient Health Questionnaire-9, mean (SD), 0=none, 27=severe depression	9.7 (5.6)
Moderate, *n* (%)	11 (28%)
Moderately severe, *n* (%)	6 (15%)
Severe, *n* (%)	2 (5%)
Generalized Anxiety Disorder-7, mean (SD), 0=none, 10=severe anxiety disorder	7.7 (5.6)
Moderate, *n* (%)	10 (26%)
Severe, *n* (%)	3 (8%)
PTSD Checklist-civilian version, mean (SD), 17=not at all severe, 85=extremely severe PTSD symptoms	40.3 (18.1)
Moderate to moderately high severity, *n* (%)	9 (23%)
High severity, *n* (%)	15 (38%)
Alcohol Use Disorders Identification Test, mean (SD), 0–12, 0=no alcohol use	2.2 (2.8)
PROMIS® (*T* score)	
Depression*, mean (SD)	55.5 (9.6)
Anxiety*, mean (SD)	58.7 (10.0)
Alcohol use, mean (SD)	46.6 (5.9)
Neuropathic pain, mean (SD)	49.0 (7.7)
Expectations of chiropractic care	
Amount of change in back pain, 0=no change, 10=complete relief	
Hoped for, mean (SD)	8.0 (1.7)
Realistically expected, mean (SD)	5.7 (2.0)
Amount of change in the impact of back pain on life, 0=no change/worse, 10=back pain no longer impacts my life	
Hoped for, mean (SD)	8.1 (1.8)
Realistically expected, mean (SD)	5.6 (1.9)

*higher score worse

### Feasibility outcomes

Most participants (*n*=25, 63%) were recruited from a pool of patients already referred to routine chiropractic care. An additional 3 were referred to the study by providers. Twenty-two individuals who received the invitational letter responded for information and were phone screened, with 5 enrolled; 14 others asked to be taken off our list. Of the 5 enrolled from this strategy, 3 were female and 1 Hispanic. Thirteen contacted us after seeing informational materials, of which 7 were enrolled. Of those who were excluded at the phone screen, 14 (28%) had received chiropractic care within 90 days and 7 (14%) had LBP that was not chronic (Fig. 1).

Scheduling was done per clinic protocol with VA staff. The mean number of DC visits over 10 weeks was 4.5 (range 1 to 7). Three participants had only 1 visit and canceled subsequent visits. Eighteen participants canceled at least 1 visit, but over half rescheduled. One participant withdrew from the trial at week 8 due to circumstances unrelated to the study.

Seven participants did not complete the second component of the baseline outcomes and 10 did not complete the HEAL instrument after the first chiropractic visit. During the first 2 months of follow-up data collection, only 6/15 completed the week 10 assessment. We then refined our reminder protocols and changed the frequency the study coordinator contacted the participant during the 5- and 10-week assessment windows from at least once to 3 times. Based on these changes, the last 24 participants completed the 10-week assessment. Our final completion rates were 33/40 (83%) for the 5-week assessment and 32/40 (80%) for the 10-week assessment (Fig. 1). Three participants completed a partial 10-week assessment via CATI. The final HEAL questionnaire was supposed to be completed prior to the last scheduled chiropractic visit. However, it was difficult to obtain this visit date and we only sent an email link to this questionnaire to 14 participants, 9 of whom completed it.

**Fig. 2:**
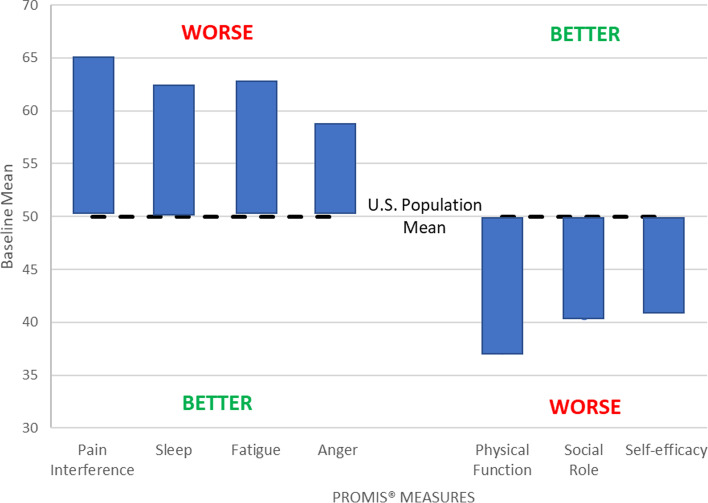
Select PROMIS® measures at baseline

The median number of minutes for participants to complete each assessment is shown in Table 3. The baseline assessments took the longest to complete, followed by the week 10 assessment. The HEAL instrument administered after the first visit took a median of 4 min to complete. The 3-item PEG tool took a median of 1 min to complete at both weeks 3 and 7. Both the second baseline and week 10 assessments had outliers who took more than 1 h to complete them.

**Table 3:** Time to complete each assessment

	Baseline visit		After first DC visit	Week 3	Week 5	Week 7	Before last DC visit	Week 10
	In-person	After						
Completed (# of participants)	40	33	30	35	33	29	9	32
Time to complete (min), median (range)	12.5 (7–25)	31 (14–7)8	4 (2–36)	1 (0–3)	28 (12–57)	1 (0–4)	1 (1–3)	38 (14–68)

Note: 0 in range indicates less than 1 min

*Abbreviation*: *DC* Doctor of chiropractic

Descriptive statistics of outcome measures over time are shown in Table 4. The mean (SD) change from baseline to week 10 on the RMDQ was 3.6 (6.1). The mean BPI pain intensity on average at baseline was lower than the DVPRS mean current pain and the PASTOR mean pain in the past 7 days and all improved over time, but only 33% of participants completed the DVPRS current pain items. The mean BPI and DVPRS pain interference measures differed similarly to their pain measures. Mean PROMIS® domains at baseline were consistent with chronic pain populations (Tables 2 and 4, Fig. 2) and improved over time (Table 4). The mean (SD) change from baseline to week 10 on the PROMIS pain interference domain was 3.6 (5.6).

**Table 4:** Outcome measures over time

Outcome measure	Baseline	Week 5	Week 10
	Mean (*SD*)	Mean (*SD*)	Mean (*SD*)
Roland-Morris Disability Questionnaire Score (0–24, 0=no disability, 24=severe disability)	12.9 (5.1)	10.2 (5.5)	9.6 (6.2)
BPI Pain Intensity (0–10, 0=no pain, 10= pain as bad as you can imagine)	4.8 (1.7)	4.7 (1.9)	4.3 (2.0)
BPI Pain Interference (0–10, 0=does not interfere, 10=completely interferes; over the past 24 h)	4.6 (2.2)	4.5 (2.0)	4.1 (2.1)
DVPRS Current Pain (0–10, 0=no pain, 10 = as bad as it could be, nothing else matters)	5.2 (2.5)	4.8 (1.7)	3.4 (2.1)
DVPRS Pain Interference (0=does not interfere, 10=completely interferes; over the past week)	5.4 (2.0)	5.0 (2.0)	4.0 (2.1)
PASTOR Pain Average (0–10, 0=no pain, 10=as bad as it could be, nothing else matters; in the past 7 days)	5.4 (1.4)	4.8 (1.8)	3.9 (2.0)
PROMIS® (*T* score)			
Pain interference*	64.4 (4.4)	61.3 (4.8)	60.5 (5.5)
Physical function	37.5 (5.1)	38.7 (5.6)	39.7 (7.6)
Fatigue*	62.3 (7.3)	61.0 (7.8)	60.9 (7.4)
Sleep disturbance*	61.8 (9.4)	62.0 (8.3)	59.8 (10.9)
Satisfaction with social role	40.8 (5.3)	40.1 (5.0)	42.4 (7.3)
Anger*	56.7 (9.0)	53.9 (10.0)	54.3 (9.7)
Self-efficacy for managing symptoms	41.6 (6.4)	N/A	43.6 (8.2)

*higher score worse; Abbreviations: *BPI* Brief Pain Inventory, *DVPRS* Defense & Veterans Pain Rating Scale, *PASTOR* Pain Assessment Screening Tool and Outcomes Registry

All HEAL items administered after the first treatment had means above the U.S. population ranging from 50.9 for spirituality to 60.9 for patient-provider connection. The HEAL mean patient-provider connection before the last treatment increased to 65.2, but was only obtained for 9 participants. Participants who completed the second baseline and week 10 assessments responded to the back-pain self-care questions.

### Adverse events

There were no serious adverse events reported during the study. Six participants self-reported discomfort following chiropractic treatment: 2 had discomfort that prevented some regular activities, 2 modified regular activities, and 1 had no change in activities. Five of those also reported soreness or increased pain lasting between a few hours to 1 week; 1 also reported dizziness; and 1 reported back crepitus.

## Discussion

Pilot studies of full-scaled clinical trials are informative, especially when a trial will take place in large, complex healthcare systems, such as VA. They can also play a key role in the planning of innovative approaches such as pragmatic trials [62]. Our pilot study allowed us to assess the feasibility of conducting a pragmatic trial of chiropractic care in a VA environment. Using the pilot data and lessons learned, we modified and refined a protocol for a full-scale, multisite, pragmatic randomized trial of multimodal chiropractic care for veterans with chronic LBP [63].

We successfully recruited our goal of 40 participants over 6 months at a rate of 6–7 per month. Our most successful recruitment method was enrolling patients already referred to routine chiropractic care. Although we enrolled only 2.5% of veterans we sent invitational letters to, these included both female and minority veterans, supporting the need for multiple recruitment strategies to increase participant diversity. Twenty-three percent of participants were female, exceeding our goal of 20%. We were able to enroll 21% of all contacts.

Eligibility criteria were broad to reflect the pragmatic design. The most common reason veterans were excluded during the phone screen was the use of chiropractic care within the past 90 days (28%). We used this exclusion because we did not want to disrupt the continuity of care. We broadened this for the full-scale trial to not being under current chiropractic care. No patients were excluded at the baseline or DC screening visits, supporting the pragmatic intent of the trial, although one veteran declined to participate at the DC visit.

The participants’ age distribution was similar to that of veterans with pain, although our sample was slightly younger [64]. Veterans have been reported to have high levels of PTSD, depression, anxiety, and alcohol use disorders, especially in younger age cohorts [65]. Most of our participants had these mental health comorbidities, except for alcohol use disorder, which provides further evidence that these recruitment strategies will connect with veterans who have chronic pain for future pragmatic trials.

We judged retention through both adherence to the treatment plan and completion rates of the outcome assessments. The mean number of visits over our 10-week trial was 4.5, which is consistent with the mean annual number of 5 chiropractic visits in VA [31]. Other than the 3 participants who attended only their first visit, the remainder appeared to adhere to their treatment plan. All participants completed the assessments at their in-person baseline visit, 83% completed the online baseline assessments made available after their in-person visit, 83% completed them at week 5, and 80% at week 10. We achieved our goal of at least 80% completion at week 10 but needed to enhance our reminder protocols early in the trial to achieve this goal.

Although we successfully programmed and used remote electronic data collection for previous clinical trials [9, 66, 67], this was our first time using REDCap. We had no issues using REDCap but recognize the necessity of strict reminder protocols and less assessment times and questionnaires. In the full-scale trial, the baseline assessment will only occur at the baseline visit and we will use the enhanced reminder protocol for all follow-up assessments, which will decrease participant burden. Completion of outcome assessments did not appear to be related to the number of completed or canceled visits. We also successfully piloted obtaining veteran demographics through the EHR.

One goal of chronic pain management is to reduce pain-related disability [68]. We observed high baseline values and moderate short-term improvements on the RMDQ, which will remain our primary outcome for the full-scale trial. The mean change in RMDQ was 3.6, slightly above the minimal clinically important change of 3.5 [69]. We also observed high baseline values in PROMIS® pain interference and a mean change of 3.6, which exceeded the minimal clinically important change of 2 to 3 points [70]; it will be a key secondary outcome.

With the goal of not having overlapping items and considering the time needed to complete the assessments, we chose not to use the BPI or most of PASTOR in the full-scale trial. Although some researchers use different anchors for BPI, it is validated for the past 24 h, which may be appropriate for acute LBP, but likely not chronic. In addition, the BPI has been validated for LBP, but not chronic LBP [71]. PASTOR was designed to enhance the clinical encounter and provide data for comprehensive evaluations of treatment effectiveness [43], but the length of the instrument and focus on the clinical encounter did not work well for our research purpose.

In the full-scale trial, we will use an EHR template to collect care provided at each DC visit and extract healthcare utilization, including prescription pain medications, rather than relying on self-report. We have chosen to use the legacy instruments to measure depression, anxiety, PTSD, and alcohol use. We will also administer the PROMIS® items that do not overlap with the legacy instruments through computer adaptive testing, where questions are dynamically asked based on the participant’s prior responses. We feel that separate pain interference, fatigue, sleep disturbance, and other domains, rather than averages over these domains for an overall pain interference score, will better allow us to assess the reduction of pain-related disability and interference. We will also ask PEG at each assessment and weekly via text messaging. Our choices are consistent with a rapid review of measures for patients with chronic musculoskeletal pain, which found substantial evidence for the use of RMDQ, PROMIS® pain interference, and PEG in VA [72].

A limitation of a single-arm study is that the willingness of patients to be randomly allocated to a treatment group is not assessed. However, our success in conducting randomized clinical trials in complex clinical environments within military healthcare facilities reduces this limitation [9, 66, 67].

## Conclusion

Prospective clinical trials using innovative and rigorous research methods to evaluate the effectiveness of chiropractic care for pain management in veterans experiencing LBP-related disability with comorbid mental health conditions are warranted. We demonstrated the feasibility of participant recruitment, retention, and electronic data collection for a pragmatic clinical trial of chiropractic care in a VA environment. Through careful examination of time to complete measures and choosing the most relevant measures without overlap, we reduced the number of outcome measures by half for the full-scale trial. Using the pilot data and lessons learned, we modified and refined a protocol for a full-scale, multisite, pragmatic, National Institutes of Health-funded randomized trial of multimodal chiropractic care for veterans with chronic LBP that began recruitment in February 2021.

## Supplementary Information

**Additional file 1.** CONSORT 2010 checklist of information to include when reporting a pilot or feasibility trial.

**Additional file 2.** ICD-10 codes for low back pain.

## Data Availability

The data are available from the corresponding author on reasonable request. For information about the dataset used in this study, please contact the Office of Data Management and Biostatistics, Palmer Center for Chiropractic Research, Palmer College of Chiropractic, Davenport, IO, at palmer-research@palmer.edu.

## Abbreviations

AE: Adverse event
AUDIT: Alcohol Use Disorders Identification Test
BPI: Brief Pain Inventory
CATI: Computer-assisted telephone interview
COCOV: Care Outcomes for Chiropractic Outpatient Veterans
DC: Doctor of chiropractic
DVPRS: Defense & Veterans Pain Rating Scale
EHR: Electronic health record
EXPECT: Expectations for Complementary and Alternative Medicine Treatments
GAD-7: Generalized Anxiety Disorder-7
HEAL: Healing Encounters and Attitudes List
IA: Iowa
ICD-10: International Classification of Diseases, edition 10
IRB: Institutional review board
LBP: Low back pain
NRS: Numerical rating scale
PASTOR: Pain Assessment Screening Tool and Outcomes Registry
PCL-C: PTSD Checklist-Civilian
PEG: Pain, Enjoyment, and General Activity
PHQ-9: Patient Health Questionnaire-9
PROMIS®: Patient-Reported Outcomes Measurement Information System
PTSD: Post-traumatic stress disorder
REDCap: Research Electronic Data Capture
RMDQ: Roland-Morris Disability Questionnaire
U.S.: United States of America
VA: U.S. Department of Veterans Affairs, Veterans Health Administration
