# Supplementary material for: Care Outcomes for Chiropractic Outpatient Veterans (COCOV): a single-arm, pragmatic, pilot trial of multimodal chiropractic care for U.S. veterans with chronic low back pain
Source: Pilot Feasibility Stud. 2022 Mar 7;8:54. doi: 10.1186/s40814-022-01008-0 (PMC8900358; doi:10.1186/s40814-022-01008-0)
Supplement: Supplementary file 2 — Additional file 2. ICD-10 codes for low back pain. [file 40814_2022_1008_MOESM2_ESM.docx]

**Additional file 2. ICD-10 codes for low back pain**

G57.00 lesion of sciatic nerve, unspecified lower limb

G57.01 lesion of sciatic nerve, right lower limb

G57. 02 lesion of sciatic nerve, left lower limb

M41.05 scoliosis, thoracolumbar

M41.06 scoliosis, lumbar

M41.07 scoliosis, lumbosacral

M41.08 scoliosis, sacral and sacrococcygeal

M41.25 other idiopathic scoliosis, thoracolumbar

M41.26 other idiopathic scoliosis, lumbar

M41.27 other idiopathic scoliosis, lumbosacral

M41.45 neuromuscular scoliosis, thoracolumbar

M41.46 neuromuscular scoliosis, lumbar

M41.47 neuromuscular scoliosis, lumbosacral

M41.85 scoliosis (other), thoracolumbar region

M41.86 scoliosis (other), lumbar region

M41.87 scoliosis (other), lumbosacral region

M41.9 scoliosis (other), unspecified

M43.05 spondylolysis, thoracolumbar region

M43.06 spondylolysis, lumbar region

M43.07 spondylolysis, lumbosacral region

M43.08 spondylolysis, sacral and sacrococcygeal region

M43.09 spondylolysis, multiple sites in spine

M43.15 spondylolisthesis, thoracolumbar region

M43.16 spondylolisthesis, lumbar region

M43.17 spondylolisthesis, lumbosacral region

M43.18 spondylolisthesis, sacral and sacrococcygeal region

M43.19 spondylolisthesis, multiple sites in spine

M43.25 fusion of spine, thoracolumbar region

M43.26 fusion of spine, lumbar region

M43.27 fusion of spine, lumbosacral region

M43.28 fusion of spine, sacral and sacrococcygeal region

M43.8X5 other unspecified dorsopathies, thoracolumbar region

M43.8X6 other unspecified dorsopathies, lumbar region

M43.8X7 other unspecified dorsopathies, lumbosacral region

M43.8X8 other unspecified dorsopathies, sacral and sacrococcygeal region

M43.8X9 other unspecified dorsopathies, site unspecified

M43.9 other unspecified dorsopathies

M47.15 spondylosis w myelopathy, thoracolumbar region

M47.16 spondylosis w myelopathy, lumbar region

M47.816 spondylosis w/out myelopathy or radiculopathy, lumbar region

M47.817 spondylosis w/out myelopathy or radiculopathy, lumbosacral region

M47.896 other spondylosis, lumbar region

M47.897 other spondylosis, lumbosacral region

M48.05 spinal stenosis, thoracolumbar region

M48.06 spinal stenosis, lumbar region

M48.07 spinal stenosis, lumbosacral region

M48.08 spinal stenosis, sacral and sacrococcygeal region

M48.25 kissing spine, thoracolumbar region

M48.26 kissing spine, lumbar region

M48.27 kissing spine, lumbosacral region

M51.25 other intervertebral disc displacement, thoracolumbar region

M51.26 other intervertebral disc displacement, lumbar region

M51.27 other intervertebral disc displacement, lumbosacral region

M51.36 other intervertebral disc degeneration, lumbar region

M51.37 other intervertebral disc degeneration, lumbosacral region

M53.3 sacrococcygeal disorder nec

M53.80 other specified dorsopathies, site unspecified

M53.86 other specified dorsopathies, lumbar region

M54.00 panniculitis affecting regions of neck and back, site unspecified

M54.16 radiculopathy, lumbar region

M54.17 radiculopathy, lumbosacral region

M54.30 sciatica, unspecified side

M54.31 sciatica, right side

M54.32 sciatica, left side

M54.30 sciatica, unspecified side

M79.1 myofascial pain

M99.03 segmental & somatic dysfunction, lumbar region

M99.04 segmental & somatic dysfunction, sacrum

Q76.415 congenital kyphosis, thoracolumbar

Q76.426 congenital lordosis, lumbar

Q76.49 other congenital malformations spine, not assoc with scoliosis

S33.5xx_ sprain of ligaments of lumbar spine

S33.6xx_ sprain of sacroiliac joint

S33.8xx_ sprain of other parts of lumbar spine and pelvis

S39.012_ strain of muscle, fascia, and tendons of lower back

S39.013_ strain of muscle, fascia, and tendon of pelvis
